# Stereoselectivity of In Vivo Processes and Bioactivity of Farrerol Enantiomers

**DOI:** 10.3390/molecules30092038

**Published:** 2025-05-03

**Authors:** Lirong Chen, Tang Yan, Dongting Huang, Wei Xu, Yongjing Liu, Xiaoying Wang, Hua Li

**Affiliations:** Institute of Structural Pharmacology & TCM Chemical Biology, College of Pharmacy, Fujian University of Traditional Chinese Medicine, Fuzhou 350122, China; clr1183@163.com (L.C.); 17684061023@163.com (T.Y.); h3315419773@163.com (D.H.); 2000017@fjtcm.edu.cn (W.X.); 2022041@fjtcm.edu.cn (H.L.)

**Keywords:** farrerol enantiomers, stereoselective, in vivo processes, antiproliferative target, HT-29 cell

## Abstract

Farrerol, a bioactive compound found in Folium Rhododendri daurici, demonstrates various biological and pharmacological effects. Nevertheless, the stereoselectivity of in vivo processes and bioactivity between its enantiomers have not been thoroughly investigated. This study aimed to explore the stereoselectivity and pharmacological activity variations in farrerol enantiomers, focusing on stereoselective pharmacokinetics, tissue distribution, in vitro metabolism using liver microsomes, in vivo intestinal absorption, molecular simulations of binding affinity with antiproliferative target, and cell viability assessed through the CCK-8 assay. The findings indicated that the pharmacokinetic characteristics of farrerol in rats’ plasma, liver, and kidney tissues displayed enantioselectivity after intragastric administration. Then, no chiral transformation between farrerol enantiomers was observed in the rat plasma when (+)-farrerol and (−)-farrerol were orally administered. Additionally, there are notable stereoselective differences in the inhibition of CYP 1A2, CYP 2C9, CYP 2C19, and CYP 3A4/5 enzymes by (+)-farrerol and (−)-farrerol (*p* < 0.01). These differences may contribute to the stereoselectivity observed in the hepatic metabolism of the two enantiomers of farrerol. In addition, there were selective differences in the binding of farrerol enantiomers to anti-proliferative targets, including UCHL3, STAT3β, PTP1B, and GSK3β. Farrerol enantiomers exhibited similar growth inhibitory effects in HT-29 cell. Overall, our work will provide a solid theoretical basis and experimental reference for the further development and utilization of farrerol enantiomers.

## 1. Introduction

Farrerol, chemically known as 2,3-dihydro-5,7-dihydroxy-2-(4-hydroxyphenyl)-6,8-dimethyl-4-benzopyrone, is a key component of Folium Rhododendri daurici [1], which is a famous traditional Chinese medicine for treating symptoms of cough, gasp, and excessive phlegm caused by acute or chronic bronchitis for thousands of years in China. Farrerol has been proven to be one of the main active ingredients for Folium Rhododendri daurici, which is often used in the treatment of respiratory diseases caused by chronic bronchitis, such as cough and excessive phlegm. Modern pharmacological studies have shown that farrerol also possesses effects, including anti-oxidation, inhibition of lung adenocarcinoma cells, improvement of chronic kidney disease, and anti-liver toxicity [2,3,4,5,6].

**Figure 1:**
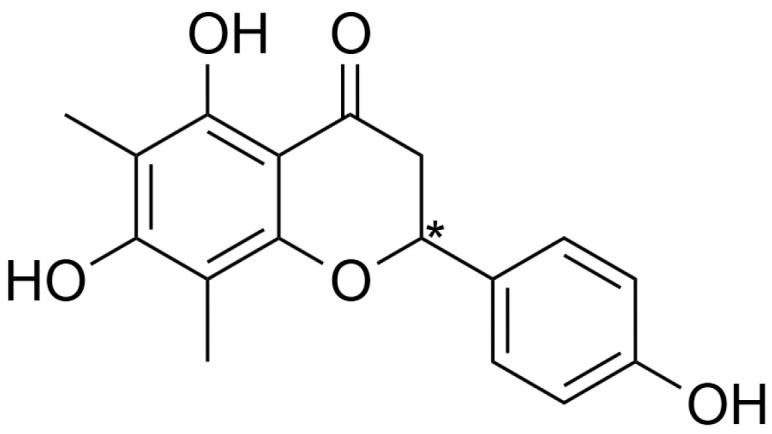
Chemical structure of farrerol. Note: “*” represents the chiral center.

Farrerol (Figure 1) has an asymmetric center in its molecular structure and thus consists of a pair of enantiomers, (−)-farrerol and (+)-farrerol. Although the enantiomers of chiral drugs have similar physical and chemical properties, it is widely known that pharmacokinetic behaviors, pharmacodynamic activity, and even the toxic effects of individual stereoisomers of chiral drugs can differ significantly. Because many endogenous macromolecular substances in the body, such as enzymes, carriers, receptors, plasma proteins, and polysaccharides, may be stereoselectively bound to enantiomers [7,8,9,10,11,12,13,14,15,16,17]. At the same time, the pharmacokinetic processes of enantiomers are relatively complex and display a stereoselective manner of absorption, distribution, metabolism, and excretion. The stereoselectivity of the process in vivo may result in different blood or plasma concentration vs. time profiles, tissue distributions, and metabolism processes of the two enantiomers, which ultimately contribute to the different pharmacological activities, toxicity, or adverse effects [18,19,20,21]. As a result, pharmacokinetic parameters obtained only in the form of racemates of chiral drugs cannot fundamentally clarify the relationship between drug concentration and action. Consequently, the stereoselectivity in metabolism and pharmacokinetic characteristics of chiral drugs plays a crucial role in assessing their therapeutic uses and potential toxic effects.

To date, extensive research has been conducted on the pharmacological properties, quality control, and drug metabolism of farrerol, yet the variations among its enantiomers have not been adequately addressed. Recently, the study of enantioseparation for rac-farrerol using high-speed counter-current chromatography was reported, which is the only report focusing on farrerol enantiomers. In our previous research, the HPLC method of chiral separation and analysis of farrerol was established using a CHIRALPAK OJ-RH column under reversed-phase conditions [22]. Moreover, farrerol has been extensively reported to interact with multiple biological targets, demonstrating its broad pharmacological potential, such as UCHL3, Stat3, PTPN1, and GSK-3β [4,19,23,24,25]. Nevertheless, the variations in pharmacological activities between the farrerol enantiomers remain undetermined.

Therefore, we investigated the stereoselectivity, pharmacokinetics, and tissue distribution of (+)-farrerol and (−)-farrerol. In addition, the main reasons for stereoselectivity in oral absorption of (+)-farrerol and (−)-farrerol were investigated by studying the metabolism in vitro liver microsomes and in vivo intestinal absorption in rats of farrerol enantiomers. Finally, the binding ability between farrerol enantiomers and the antiproliferative target was calculated by molecular docking techniques. We also used the CCK-8 assay to evaluate the cell viability of HT-29 cells. So far, there has been no literature about the stereoselectivity and differences in pharmacological activities of farrerol enantiomers, including a stereoselective pharmacokinetics study in vivo, metabolism in vitro liver microsomes, and in vivo intestinal absorption. As a result, our research will enhance the understanding of the adsorption, metabolic behavior, and variations in pharmacological activities of individual farrerol enantiomers in vivo. Moreover, our work will provide a solid theoretical basis and experimental reference for the further development and utilization of farrerol enantiomers.

## 2. Results and Discussion

### 2.1. Preparation and Characterization of Farrerol Enantiomers

**Figure 2:**
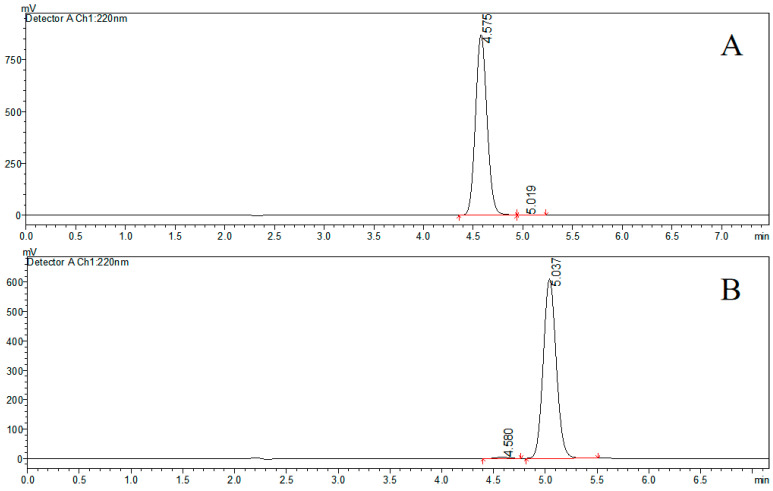
HPLC chromatograms of (+)-farrerol (**A**) and (−)-farrerol (**B**).

The specific optical rotation data are presented in Table 1. The HPLC chromatographic profile (Figure 2) demonstrated baseline separation of the enantiomers, with retention times of 4.575 min for (+)-farrerol and 5.037 min for (−)-farrerol. Purity assessment by the normalized peak area method yielded enantiomeric purities of 99.76% for (+)-farrerol and 99.40% for (−)-farrerol, respectively.

### 2.2. Stereoselective Pharmacokinetics Study in Rat Plasma

**Figure 3:**
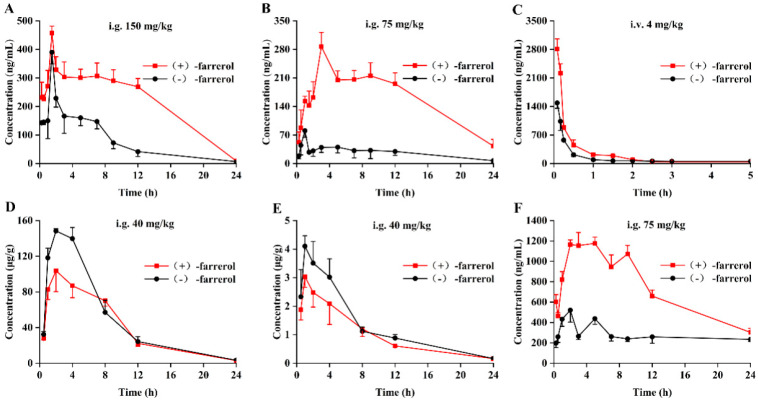
Mean concentration-time profiles of farrerol enantiomers in rat plasma and tissues. (**A**): Oral administration of 150 mg/kg farrerol racemate, rat plasma (*n* = 5). (**B**): Oral administration of 75 mg/kg farrerol racemate, rat plasma (*n* = 5). (**C**): Intravenous administration of 4 mg/kg farrerol racemate, rat plasma (*n* = 5). (**D**): Oral administration of 40 mg/kg farrerol racemate, rat liver tissue (*n* = 6). (**E**): Oral administration of 40 mg/kg farrerol racemate, rat kidney tissue (*n* = 6). (**F**): Oral administration of 75 mg/kg farrerol isomers, rat plasma (*n* = 5).

The newly established stereoselective bioanalytical method was utilized to quantify (+)-farrerol and (−)-farrerol in the plasma of Sprague–Dawley rats following intravenous administration of 4 mg/kg and oral administration of 75 and 150 mg/kg of the farrerol racemate. The doses of 150 mg/kg and 75 mg/kg for intragastric administration were selected based on preliminary pharmacological studies demonstrating the effective dose range of farrerol in rat models [3,26,27,28], and the 4 mg/kg intravenous dose was determined through dose-conversion calculations from effective oral doses, accounting for bioavailability differences between administration routes [29]. The pharmacokinetic data were optimally characterized by a noncompartmental model. The mean concentration-time profiles for the farrerol enantiomers are illustrated in Figure 3, along with the corresponding key pharmacokinetic parameters presented in Table 2. As seen from Figure 3C and Table 2, the data obtained in rats after an intravenous administration of 4 mg/kg farrerol racemate demonstrated that the pharmacokinetic profile of (+)-farrerol was similar to that of (−)-farrerol. The C_max_ of (+)-farrerol was 2662.74 ng/mL, which was significantly higher than that of (−)-farrerol (*p* < 0.01). As for AUC_0–24_, although the value of (+)-farrerol was slightly higher, there was no significant difference compared with (−)-farrerol.

Concerning oral administration of the farrerol racemate, the results of Figure 3A,B and Table 2 indicated that there was a significant stereoselective difference in the plasma pharmacokinetic behavior of the farrerol enantiomer in SD rats (*p* < 0.05). As directly observed in Figure 3B, after oral administration of 75 mg/kg farrerol racemate, the concentration of the two enantiomers of farrerol in rat plasma increased rapidly after administration. The time-to-peak concentration (T_max_) of (−)-farrerol was reached at 1 h, which was faster than (+)-farrerol, indicating that stereoselectivity existed in the process of absorption. By contrast, the C_max_ values and the AUC_0–t_ of (+)-farrerol were 287 ng/mL and 2399 ng/mL*h, which were about 3.5- and 5.6-fold higher than that of (−)-farrerol, respectively, demonstrating that (+)-farrerol showed greater plasma concentration than (−)-farrerol. Moreover, the elimination t_1/2_ of (+)-farrerol was 19.2 h, 1.5 times longer than that of (−)-farrerol, proving that a significant difference exists in the elimination phase between the two enantiomers. When orally administered at 150 mg/kg, the same trend of enantioselectivity of the two enantiomers was observed as that of orally administered at 75 mg/kg, in which the values of C_max_ and AUC_0–t_ of (+)-farrerol were higher than those of (−)-farrerol.

In conclusion, statistical analysis revealed significant differences between the two enantiomers regarding C_max_, AUC, MRT, and t_1/2_. Additionally, the concentration of (+)-farrerol in rat plasma consistently exceeded that of (−)-farrerol. These findings indicate notable stereoselectivity in the pharmacokinetic profiles of (+)-farrerol and (−)-farrerol in rats, with (+)-farrerol demonstrating superior bioavailability compared to its enantiomer.

To make the mechanism of the stereoselective pharmacokinetics of farrerol clearer, the pharmacokinetics study of single enantiomers was carried out after oral administration of 75 mg/kg of (+)-farrerol and (−)-farrerol, respectively. The blood collection time and plasma pretreatment method were consistent with those of racemic administration. The average drug concentration-time profiles, along with the relevant pharmacokinetic parameters, are presented in Figure 3F and Table 2. It was revealed from the results that the pharmacokinetic behavior of the two enantiomers in rat plasma was inconsistent. It was observed that (−)-farrerol reached C_max_ at t_max_ 2 h, with a peak concentration that was only one-third of that achieved by (+)-farrerol at its t_max_. Nevertheless, (+)-farrerol posed a higher concentration in rat plasma, and the C_max_ values and the AUC_0–t_ of (+)-farrerol were 1174 ng/mL and 117,593.5 ng/mL*h, which were about 2.55- and 2.61-fold higher than that of (−)-farrerol, respectively. It is important to highlight that neither of the farrerol enantiomers experienced chiral transformation. Notably, in single-enantiomer administration, the similar half-lives of (+)-farrerol and (−)-farrerol (Table 2) contrast with the divergent values observed in racemate administration. This suggests that enantiomer-enantiomer interactions may occur when both forms are co-administered (e.g., competition for metabolic enzymes or transporters), leading to altered distribution or clearance kinetics. In contrast, when administered as a farrerol isomer, the absence of such interactions allows each enantiomer to exhibit its intrinsic elimination rate, resulting in comparable half-lives. Further studies are needed to elucidate the specific mechanisms underlying these stereoselective effects.

### 2.3. Stereoselective Pharmacokinetics Study in Rat Liver and Kidney

**Figure 4:**
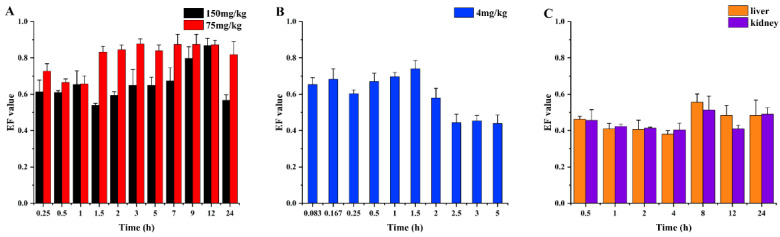
Dynamic changes in the EF values of farrerol in rat plasma after i.g. (**A**), rat plasma after i.v. (**B**), and rat liver and kidney after i.g. 40 mg/kg (**C**).

The findings presented in Figure 4 and Table 3 indicate that the concentration of both farrerol enantiomers in liver tissue increased significantly following oral administration. Both enantiomers reached their C_max_ at 2 h, suggesting that the absorption characteristics of the farrerol enantiomers in the liver are comparable. Nevertheless, the C_max_ and AUC_0–24_ values of (−)-farrerol were 148.4 µg/g and 1200.9 µg/g*h, which were 1.43 and 1.23 times higher than those of the (+)-farrerol, respectively. This phenomenon indicates that (−)-farrerol has a higher accumulation in the liver than (+)-farrerol, maybe resulting in a lower concentration in plasma. Moreover, the concentration of the farrerol enantiomers in the liver tissues decreased to a lower level after 12 h of oral administration, and until 24 h, the elimination process of the two enantiomers of farrerol in the liver showed a consistent behavior.

As for the two enantiomers in the kidney, the curve of the mean concentration of farrerol enantiomers over time presented consistent stereoselective tendencies with that in the liver. It was found that (−)-farrerol showed a higher exposure than (+)-farrerol, and the C_max_ and AUC_0–24_ values of (−)-farrerol were 1.36 and 1.20 times higher than those of (+)-farrerol. It is worth noting that the concentration of the farrerol enantiomers in the liver tissue is much higher than in the kidney, indicating the liver tissue is the main metabolic organ of farrerol, which is consistent with those in the tissue distribution report of rac-farrerol.

The stereoselective alterations of the farrerol in rat plasma, liver, and kidney tissue were evaluated by determination of the enantiomeric fraction (EF). The EF value is calculated according to the following formula: EF = E(+)/(E(+) + E(−)), where E(+) and E(−) denote the peak areas of the (+)-farrerol and (−)-farrerol, respectively. The EF value of racemic farrerol is 0.5. The EF values of racemic farrerol in plasma, liver, and kidney tissue after oral and intravenous administration are shown in Figure 4, which was used to investigate the changes and transformations of the two enantiomers in plasma, liver, and kidney tissue. As seen from Figure 4B, after intravenous administration, the EF value of farrerol enantiomers in rat plasma almost always fluctuated around 0.7, as well as the (+)-farrerol is slightly more than the (−)-farrerol within 1.5 h. In addition, the elimination rates of (+)-farrerol and (−)-farrerol were similar during the sampling period. Oral administration is shown in Figure 4A, EF values were all greater than 0.6, especially for the dose of 75 mg/g, where the EF value fluctuated around 0.8, ranging from approximately 1.5 to 24 h. The results demonstrated that (+)-farrerol was the superior enantiomer in rat plasma when taken orally. As shown in Figure 4C, the EF values of the farrerol enantiomers in rat liver and kidney tissues were less than 0.5, meaning that the levels of (−)-farrerol were greater than those of (+)-farrerol in liver and kidney tissues within 24 h.

### 2.4. Stereoselective In Vitro Metabolism of Liver Microsomal

The CYP450 enzyme subtypes involved in the metabolism of (+)-farrerol and (−)-farrerol were assessed using chemical inhibition experiments, in which the farrerol enantiomers were incubated with specific chemical inhibitors at the same time, accomplished through the use of phenacetin, diclofenac, *S*-mephenytoin, dextromethorphan, midazolam, coumarin and chlorzoxazone as marker substrate of CYP1A2, CYP2C9, CYP2C19, CYP2D6, CYP3A4/5, CYP2A6 and CYP2E1, respectively.

**Figure 5:**
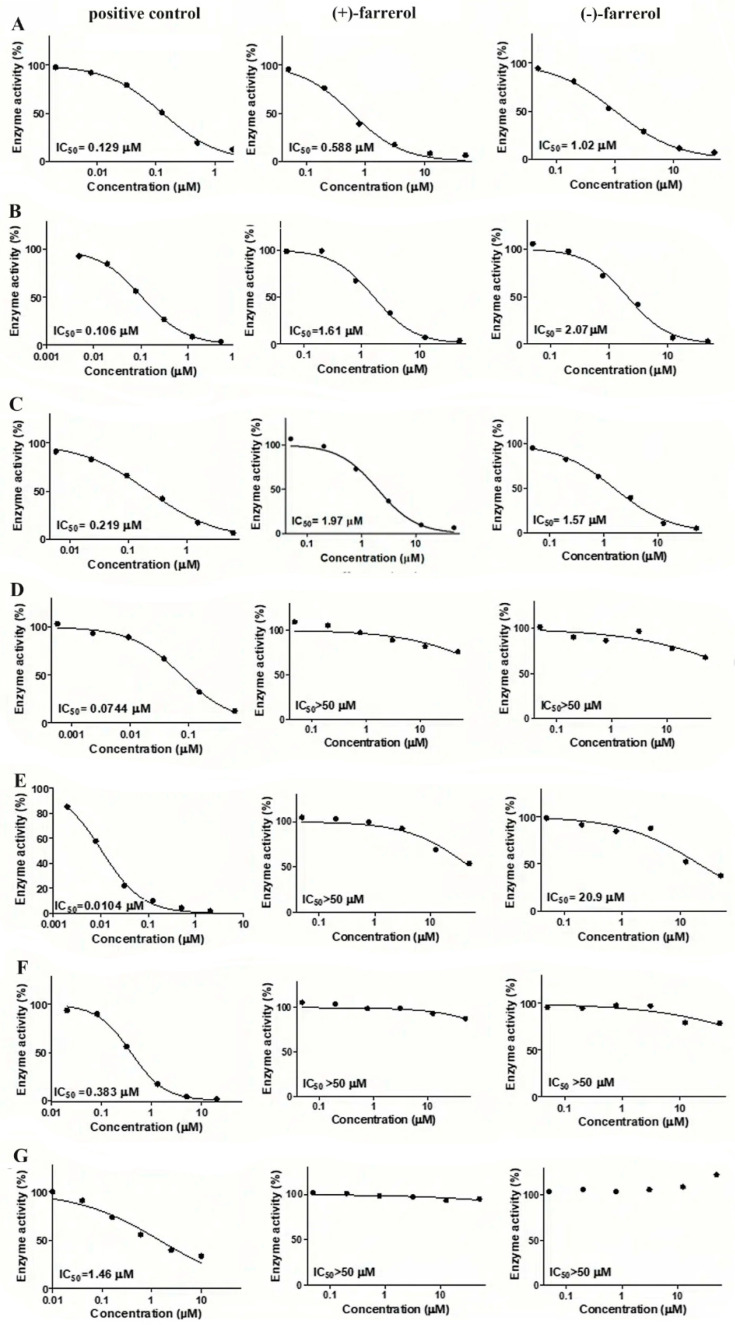
Inhibition curves of positive inhibitors, (+)-farrerol and (−)-farrerol on 7 subtypes of CYP enzyme (**A**): CYP1A2; (**B**): CYP2C19; (**C**): CYP2C9; (**D**): CYP2D6; (**E**): CYP3A4/5; (**F**): CYP2A6; (**G**): CYP2E1.

As seen in Figure 5 and Table 4, in the selected human liver P450 isoforms, CYP1A2, CYP2C9, and CYP2C19 showed activity toward (+)-farrerol, while CYP1A2, CYP2C9, CYP2C19, and CYP3A44/5 were involved in (−)-farrerol metabolism. These results indicate that the types of P450 enzyme subtypes involved in the metabolism of the two farrerol enantiomers are different. On the other hand, the inhibition intensity of the two enantiomers of farrerol on P450 enzyme subtypes was also different. In the studied human liver P450 isoforms, potent inhibition of (+)-farrerol on CYP1A2 activities was observed with *IC*_50_ values of 0.588 µmol/L. Moderate inhibition of (+)-farrerol on CYP2C9 and CYP2C19 was shown with IC 50 values of 1.97 and 1.61 µmol/L, respectively. For (−)-farrerol, moderate inhibition was observed against CYP1A2 (*IC*_50_ = 1.02 µmol/L), CYP2C9 (*IC*_50_ = 1.57 µmol/L), and CYP2C19 (*IC*_50_ = 2.07 µmol/L), while weaker inhibition was shown against CYP3A4/5 (*IC*_50_ = 20.9 µmol/L). In conclusion, there are some stereoselective differences between (+)-farrerol and (−)-farrerol in the inhibition of CYP 1A2, CYP 2C9, CYP 2C19, and CYP 3A4/5 enzymes (*p* < 0.01), which may lead to stereoselectivity in liver metabolism between the two enantiomers of farrerol.

### 2.5. Stereoselectivity of Intestinal Absorption of Farrerol Enantiomers

The absorption rate constant (K_a_) and permeability coefficient (*P*_eff_) were calculated using the following equation:

$$K_{a}=\left(1-C_{2}V_{2}/C_{1}V_{1}\right)\times V/\pi r^{2}l$$

$$P_{eff}=-V/2\pi rl\times ln{(C}_{2}V_{2}/C_{1}V_{1})$$

where V_1_ is the volume of perfusion liquid, V_2_ is the volume of receiving liquid, V is the perfusion velocity, C_1_ is the mass concentration of (+)-farrerol and (−)-farrerol in the perfusion liquid, C_2_ is the mass concentration of (+)-farrerol and (−)-farrerol in the receiving liquid, r and l are the radius and length of the perfusion intestinal segment, respectively.

The data of K_a_ and *P*_eff_ of (+)-farrerol and (−)-farrerol in the four intestinal segments are presented in Table 5. When different concentrations of farrerol enantiomers were used for rat single-pass intestinal perfusion, the main intestinal segments absorbed by farrerol enantiomers were slightly different. At a low concentration of 66 μg/mL, the absorption of farrerol enantiomers from various intestinal segments follows the sequence: duodenum > jejunum > ileum > colon. At high concentrations, the permeability of (+)-/(−)-farrerol was observed to follow this order: duodenum > jejunum > colon > ileum. In the middle concentration, the permeability of (+)-/(−)-farrerol appeared best in the duodenum. It is important to highlight that there were no statistically significant differences in the values of K_a_ and *P*_eff_ between (+)-farrerol and (−)-farrerol. This suggests that the absorption of both enantiomers in the rat small intestine was not stereoselective within the concentration range examined. Table 5 presents the K_a_ and *P*_eff_ of (+)-/(−)-farrerol (132 μg/mL) of different segments of rat intestine with and without verapamil. In conclusion, the K_a_ and *P*_eff_ values for both (+)-farrerol and (−)-farrerol were significantly increased (*p* < 0.05) in intestinal segments other than the ileum when verapamil was present, compared to the values obtained in the absence of the P-glycoprotein inhibitor. This suggests that the farrerol enantiomers may function as substrates for P-glycoprotein in rats. However, there were no significant differences in *P*_eff_ and K_a_ values between (+)-farrerol and (−)-farrerol, indicating that P-gp inhibitors did not affect the absorption differences of (+)-farrerol and (−)-farrerol in the four intestinal tracts of rats.

### 2.6. Stereoselective Binding of Farrerol Enantiomers to Antiproliferative Target

**Figure 6:**
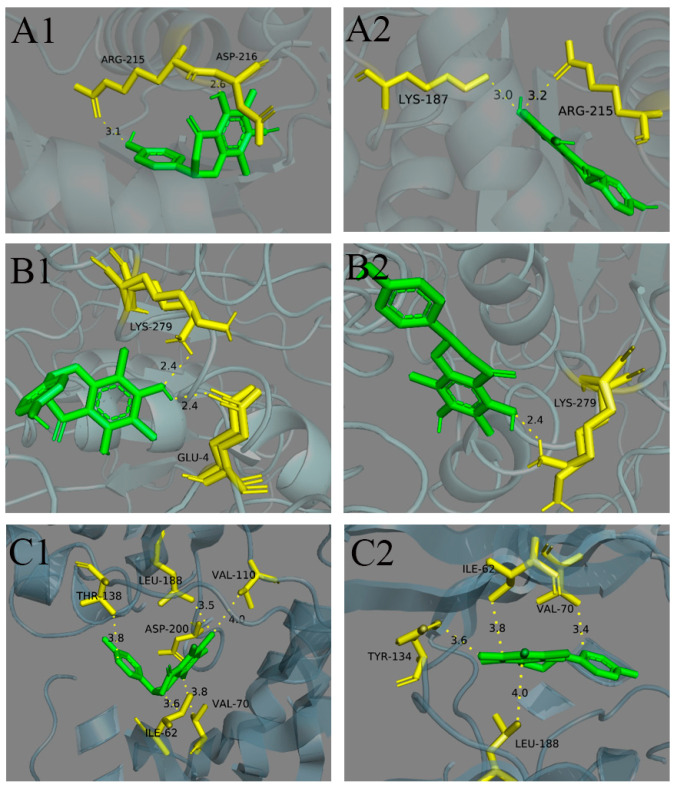
Docking results. (**A1**). 3D view of UCHL3 in complex with (+)-farrerol. (**A2**). 3D view of UCHL3 in complex with (−)-farrerol. (**B1**). 3D view of PTPN1 in complex with (+)-farrerol. (**B2**). 3D view of PTPN1 in complex with (−)-farrerol. (**C1**). 3D view of GSK-3β (4acg) in complex with (+)-farrerol. (**C2**). 3D view of GSK-3β (4acg) in complex with (−)-farrerol. UCHL3, PTPN1, and GSK-3β are shown as carbon atoms (yellow and bluish-gray), and farrerol is depicted by sticks (green).

To elucidate the stereoselective binding behavior of farrerol enantiomers to potential antiproliferative targets, we performed molecular docking studies with four reported targets of farrerol: UCHL3 [24], Stat3 [4], PTPN1 [30], and GSK-3β [25]. Our findings reveal distinct binding preferences at the molecular level, highlighting the significance of chirality in farrerol’s interactions with these proteins. The results are shown in Figure 6 and Table 6. Notably, PTPN1 exhibited the lowest binding energy with farrerol, suggesting a strong and stable interaction. This result aligns with previous reports implicating PTPN1 as a plausible target for farrerol’s antiproliferative effects. In contrast, a more pronounced stereoselective difference was observed in the binding of farrerol to UCHL3, where (−)-farrerol demonstrated significantly higher binding affinity than its enantiomer. This finding underscores the critical role of stereochemistry in ligand-protein recognition, particularly for UCHL3-mediated biological activity.

For GSK-3β, we conducted docking simulations using four crystal structures (PDB IDs: 4acc, 4acd, 4acg, and 4ach). Intriguingly, the binding free energies across all four conformations were nearly identical, consistent with the findings of Chaoqun Yan et al. [25]. However, further analysis revealed a subtle but meaningful enantioselectivity, with (−)-farrerol exhibiting a preferential binding mode over its counterpart. This suggests that while GSK-3β may accommodate farrerol in multiple conformations, the (−)-farrerol remains the more favorable conformation in terms of binding efficiency.

Collectively, these results provide mechanistic insights into the stereoselective interactions of farrerol with key antiproliferative targets. The varying degrees of enantioselectivity across different targets may reflect differences in the structural flexibility and binding pocket architectures of these proteins. The superior binding affinity of (−)-farrerol to both UCHL3 and GSK-3β highlights its potential as the more biologically active enantiomer, warranting further experimental validation.

### 2.7. Stereoselective Growth Inhibition of HT-29 Cells by Farrerol Enantiomers

**Figure 7:**
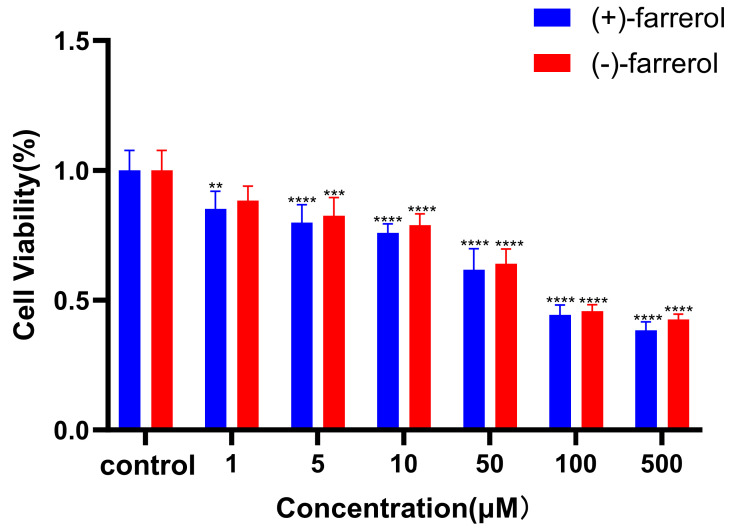
Cytotoxicity of farrerol enantiomers to HT29 cells. Note: Compared with the control group, ** *p* < 0.01, *** *p* < 0.001, **** *p* < 0.0001.

The CCK-8 assay results (Figure 7) demonstrated that both enantiomers of farrerol had growth inhibitory effects on HT-29 cells, with all treatments showing statistically significant effects compared to the control (*p* < 0.05). Furthermore, no notable difference in cytotoxicity was observed between the two farrerol isomers at any concentration, suggesting that the chiral center of farrerol does not play a critical role in its antiproliferative effects on HT-29 cells. This lack of stereoselectivity implies that the molecular targets mediating farrerol’s growth inhibition may not distinguish between enantiomers, or that both forms exhibit similar cellular uptake and metabolic stability. Further studies are needed to explore whether stereochemical differences influence other biological activities or become apparent under different experimental conditions.

## 3. Materials and Methods

### 3.1. Chemicals and Reagents

Racemic farrerol (purity: 99%) and formononetin (internal standard, IS, purity: 98%) were supplied by PUSH Bio-technology (Chengdu, China). Chiralcel OJ-RH chiral preparative column was supplied by Daicel Chiral Technologies (Shanghai, China) Co., Ltd. Chromatographic separations were performed using an Autoprep HPLC semi-preparative system (Daicel Chiral Technologies China Co., Ltd., Shanghai, China). Optical rotations were measured with an SGW-1 automatic polarimeter (Shanghai Jingke Leici, Shanghai, China) at 20 °C using the sodium D-line (589.4 nm). (+)-farrerol (purity: 98%) and (−)-farrerol (purity: 98%) are prepared by our laboratory. High-performance liquid chromatography (HPLC)-grade methanol (MeOH), acetonitrile (ACN), and formic acid (FA) were obtained from National Pharmaceutical Group Chemical Reagent Co., Ltd. (Shanghai, China). Diethyl ether (A.R.) was also provided by National Pharmaceutical Group Chemical Reagent Co., Ltd. (Shanghai, China). Deionized water was purified using the Milli-Q academic water purification system (Millipore, MA, USA). Human P450s (CYP1A2, CYP2C9, CYP2C19, CYP2D6, CYP3A4/5, CYP2A6, and CYP2E1) were purchased from CORNING Biotechnology (Shanghai, China). Krebs-Ringer’s (K-R)solution was obtained from Shanghai Yuanye Biotechnology (Shanghai, China). Verapamil (purity: 99%) was purchased by Beijing Solarbio Science & Technology (Beijing, China). CERTIFIED GOLD FETAL BOVINE SERUM was purchased from Umedium (Hefei, China). DMEM basic (1×) was purchased from Gibco (Shanghai, China). Penicillin Streptomycin Solution (100×) was obtained from NCM Biotech (Suzhou, China). Cell Counting Kit 8 was purchased from ABKBio (Xiamen, China).

### 3.2. Animals, Cells

Male Sprague–Dawley (SD) rats weighing approximately 250 ± 20 g were obtained from Shanghai Slaughter Laboratory Animal Co., Ltd., under Animal License No. SCXK (Shanghai, China) 2022-0004. They were housed at an ambient temperature of (22 ± 2) °C and a relative humidity of (50 ± 10)%. After five days of acclimation, the animals were starved overnight before the administration of the drug, but free access to autoclaved distilled water was provided. All experimental procedures were conducted under the guidelines established by the Experimental Animal Care and Use Committee at Fujian University of Traditional Chinese Medicine (Approval No. 3W2023139, Fuzhou, China).

HT-29 human colon cancer cells (IMMOCELL, Xiamen, China) were cultured in high-glucose DMEM supplemented with 10% fetal bovine serum, 100 U/mL penicillin, and 50 μg/mL streptomycin at 37 °C in a 5% CO_2_ atmosphere. They were routinely passaged using standard trypsinization procedures.

### 3.3. Preparation and Characterization of Farrerol Enantiomers

The enantiomeric (+)-farrerol and (−)-farrerol reference standards were initially prepared using a Chiralcel OJ-RH chiral preparative column, with subsequent purity evaluation conducted for both individual enantiomers. The specific optical rotation was determined using an automatic polarimeter under controlled conditions (20 °C, λ = 589.4 nm), enabling calculation of the enantiomeric excess (ee) values.

For chromatographic characterization, the prepared enantiomers were analyzed under the following optimized conditions: mobile phase: acetonitrile/water (40:60, *v*/*v*) isocratic elution; flow rate: 0.5 mL/min; column temperature: 25 °C; detection wavelength: 295 nm. Comparative retention time analysis was performed between the resolved enantiomers and racemic farrerol to establish the elution order on the Chiralcel OJ-RH column.

### 3.4. Stereoselective Pharmacokinetics Studies

#### 3.4.1. Pharmacokinetics Studies in Rat Plasma

Twenty-five rats were randomly divided into five experimental groups (A, B, C, D, and E). Groups A and B received farrerol racemate via intragastric administration at doses of 150 mg/kg and 75 mg/kg, respectively. Group C was administered 4 mg/kg of farrerol racemate through caudal intravenous administration. Groups D and E received intragastric administrations of (+)-farrerol and (−)-farrerol at a dosage of 75 mg/kg, respectively. Blood samples (300 μL) were obtained from the orbital venous plexus into heparinized tubes at various time points: 0.25, 0.5, 1, 2, 3, 5, 7, 9, 12, and 24 h following gavage administration, and at 0.083, 0.167, 0.25, 0.5, 1, 1.5, 2, 2.5, 3, and 5 h post-intravenous administration. Plasma was separated by centrifugation (3000 rpm, 10 min) and stored at −80 °C until analysis.

A total of 150 μL of plasma and 50 μL of the internal standard solution were added to a 1.5 mL EP tube and mixed using a vortex for 30 s. Subsequently, 500 μL of ether was introduced, and the mixture was vortexed for 5 min. Following centrifugation at 10,000 rpm for 10 min, the resulting organic layer was carefully transferred to a separate tube. Each sample underwent two extraction processes. The resulting ether layer was then evaporated to dryness using a continuous air stream at room temperature. A 250 μL aliquot of methanol was employed to re-dissolve the residue, utilizing vortex mixing for 2 min. Following centrifugation at 14,000 rpm for 10 min, the supernatant was subsequently injected into the HPLC-MS system for analysis.

#### 3.4.2. Pharmacokinetics Studies in Rat Liver and Kidney Tissue

Forty-two rats were divided randomly into seven groups (6 rats per time point at 0.5, 1, 2, 4, 8, 12, and 24 h) and were orally administered farrerol racemate at a dose of 40 mg/kg. Rats from each group were euthanized, and kidney and liver tissues were subsequently obtained. After washing three times using 0.9% sodium chloride solution, the attached blood and fat tissues were removed. Afterward, 0.5 g of the precisely weighed liver and kidney tissues were added to 1 mL 0.9% sodium chloride solution and homogenized in an ice bath. Subsequently, 150 µL of supernatant was transferred into a 2.0 mL EP tube for pre-treatment, in the same way as the pretreatment of plasma samples (see Section 3.4.1).

#### 3.4.3. Chiral HPLC-MS/MS Conditions

Stereoselective chromatographic analysis of farrerol enantiomers was conducted utilizing an ACQUITY UPLC I-Class UPLC system (Waters, Milford, MA, USA), which is equipped with a quaternary pump and an autosampler. The chiral separation was carried out employing a Chiralpak OJ-RH column (250 mm × 4.6 mm, 5 μm), which was obtained from Daicel Chemical Industries (Shanghai, China). The mobile phase consisted of ACN-H_2_O (60:40, *v*/*v*), and the constant flow rate was 0.5 mL/min. The column temperature was set at 25 °C, and the injection volume was 10 μL.

Mass detection was conducted using a triple quadrupole mass spectrometer (Waters Co., USA) equipped with an electrospray ionization (ESI) source. The detection of enantiomers and internal standards (IS) was carried out in multiple reaction monitoring (MRM) mode utilizing negative ionization. The *m*/*z* 299→179 for enantiomers and *m*/*z* 267→252 for IS were selected as detecting ions for quantitation. The collision energies (CE) were established at 22 eV and 20 eV, respectively. The primary mass spectrometry (MS) parameters were configured as follows: capillary voltage was set to 3.5 kV, ion source temperature was maintained at 150 °C, desolvation gas flow rate was 800 L/h, and the cone voltage (CV) was adjusted to 1.3 V.

#### 3.4.4. Methods Validation

The developed method was assessed under the bioanalytical method validation guidelines set forth by the Chinese Pharmacopeia and the US FDA. The method was assessed for its selectivity, sensitivity, linearity, accuracy, precision, and matrix effect (ME). The results are provided in the Supplementary Materials.

### 3.5. Stereoselective In Vitro Metabolism of Liver Microsomal

#### 3.5.1. Liver Microsomal Preparation and Incubation

A liver microsome working solution (20 mg/mL) contained potassium phosphate buffer (0.1 M, pH 7.4), hepatic microsomes and marker substrate solution, which phenacetin, diclofenic acid, *S*-mephenytoinwer, dextromethorphan, midazolam, coumarin and chlorzoxazone used as substrates for CYP1A9, CYP2C9, CYP2C19, CYP2D6, CYP3A4/5, CYP2A6 and CYP2E1, respectively. In brief, 238.5 µL of liver microsome working solution was placed in a 1 mL EP tube. After adding 1.5 µL (+)-farrerol, (−)-farrerol, or positive control solution of DMSO, the mixture was incubated at 37 °C for 5 min. The reaction was started by injecting NADPH (1 mM), and the resulting mixture was incubated at 37 °C for 10 min. After centrifuging at 4000 rpm for 15 min, the supernatant was transferred and analyzed using LC-MS/MS to detect the corresponding metabolites. Each incubation was conducted in triplicate (*n* = 3).

The residual activity of the enzyme was as the *Y*-axis, which was obtained by comparing the amounts of metabolites in the reaction with different concentrations of farrerol enantiomers or positive control to the amounts of metabolites in the reaction without the farrerol enantiomers or positive control, as well as the logarithmic value of the farrerol enantiomers or positive control concentration as *X*-axis to obtain the suppression curve. The values of median inhibitory concentration (*IC*_50_) of farrerol enantiomers or positive control were calculated using GraphPad Prism 10.1.2.

#### 3.5.2. HPLC-MS/MS Conditions

An API 4000 LC/tandem mass spectrometry was used to analyze the concentration of metabolites of various chemical agents using a validated mode. An Atlantis T3 column (2.1 mm × 50 mm, 5 μm) was utilized, employing mobile phases consisting of a 0.1% formic acid solution (A) and a 0.1% acetonitrile formate solution (B). The gradient elution program for detection of CYP 1A2, 2C9, 2C19, 2D6, 3A4/5, and 2A6 metabolite was as follows: 0–0.30 min, 1% B; 0.30–0.50 min, 1–25% B; 0.50–0.90 min, 25% B; 0.90–1.40 min, 25–45% B; 1.40–1.60 min, 45–99% B; 1.60–2.00 min, 99% B; 2.00–2.01 min, 99–1% B, with a constant flow rate of 0.85 mL/min throughout the analysis. As for the detection of CYP 2E1 metabolite, the Flow composition was as follows: 0–0.30 min, 10% B; 0.30–1.30 min, 10–55% B; 1.30–1.50 min, 55–95% B; 1.50–2.00 min, 95% B; 2.00–2.01 min, 95–10% B; the low rate was 0.85 mL/min. ESI was conducted in positive ion mode. MRM was utilized to quantify each metabolite, detecting the following transitions: *m*/*z* 152.1/110 for acetaminophen, 312.1/230.0 for 4′-Hydroxydiclofenac, 235.0/150.0 for 4′-hydroxymephentoin, 258.2/199.1 for Dextrorphan, 342.1/203.0 for 1′-hydroxymidazolam, 161.1/133.1 for hydroxycoumarin, and 184.0/120.0 for 6-hydroxyclozoxadone.

### 3.6. Stereoselectivity of Intestinal Absorption

#### 3.6.1. The Method of Rat Single-Pass Intestinal Perfusion

The single-pass intestinal perfusion studies in rats were conducted following previously established methodologies [31,32]. A total of 24 male Sprague–Dawley rats, each weighing between 250 and 350 g at the start of the study, were utilized and allocated into four groups (*n* = 6). These groups included a low-dose group (66.00 µg/mL), a medium-dose group (132.00 µg/mL), a high-dose group (180.00 µg/mL) of farreol racemate solution, and a group receiving the P-gp inhibitor verapamil (50.00 µg/mL). Before the surgical procedure, animals were fasted overnight with water ad libitum. Rats were anesthetized via intraperitoneal administration of 20% urethane at a dosage of 6 mL/kg and subsequently positioned on a heating pad. Their normal body temperature (37.0 ± 0.5 °C) was sustained using an infrared lamp.

A midline incision was made to open the abdomen, allowing for the exposure of 35–45 cm of the small intestine, along with a 10 cm segment comprising the duodenum, jejunum, ileum, and colon. This segment was cannulated with Teflon tubing (0.42 cm in diameter) at both the proximal and distal ends, which were then secured with silk sutures. This segment was gently flushed with warmed normal saline to remove intestinal contents. Then, farrerol racemate maintained at 37 °C was perfused through the lumen of the small intestine at a flow rate of 0.2 mL/min. After a 30 min equilibration period to reach steady-state outlet concentrations, the perfusate samples were collected every 15 min for 120 min in micro-centrifuge tubes. The length of the small intestinal segment was measured following the last collection. All collected samples were stored at −20 °C. 500 μL of collected intestinal perfusion samples were placed in an EP tube and then dried with nitrogen at 37 °C. The residue was redissolved and precipitated with 200 μL of methanol by vortex-mixing for 5 min. Following centrifugation at 13,000 rpm for 10 min, the supernatant samples were analyzed using the previously validated RP-HPLC-DAD method.

#### 3.6.2. HPLC Method and Method Validation

**Figure 8:**
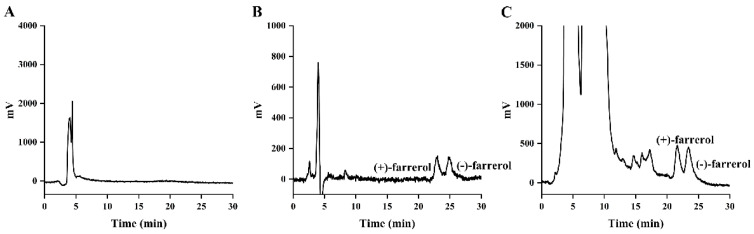
Typical HPLC chromatograms of (+)-/(−)-farrerol in (**A**) blank perfusate, (**B**) blank perfusate spiked with farrerol racemate, and (**C**) actual perfusate sample after administration of farrerol racemate.

The chiral HPLC technique employed for the analysis of farreol enantiomers in intestinal perfusate aligned with the chromatographic procedure outlined in Section 3.4.3. This method underwent validation for parameters including linearity, range, extraction recovery, specificity, precision, and accuracy. Figure 8 displays the representative chromatograms of blank perfusate, blank perfusate spiked with farrerol racemate, and a perfusate sample obtained following the administration of farrerol racemate. The results demonstrate that there was no matrix interference detected at the retention times of both (+)-farrerol and (−)-farrerol. The linearity of (+)-farrerol and (−)-farrerol in standard solutions was constructed at six different concentrations from 25.38 to 5076.40 ng/mL and 26.42–5283.60 ng/mL. The regression equations for (+)-farrerol and (−)-farrerol were determined to be y = 59.47x − 1324.22 (r = 0.9999) and y = 66.28x − 1415.14 (r = 0.9999), respectively, by plotting the peak area of the enantiomers (Y) against their concentrations (X). The RSD for the intra and inter-day precisions of three concentrations of farrerol enantiomers was all less than 3.0%; meanwhile, the mean accuracy of the two farrerol enantiomers was in the range of 88.8~99.5%, indicating that this method exhibits established reliability and accuracy. The extraction recoveries of farrerol in intestinal perfusate at three different concentration levels ranged from 90.57% to 95.57%.

### 3.7. Stereoselective Binding of Farrerol Enantiomers to UCHL3

The 3D structures of (+)-farrerol and (−)-farrerol were downloaded from PubChem. Crystal structure of human UCHL3 in complex with Farrerol (PDB ID: 7YV4) [24], transcription factor STAT3β/DNA complex (PDB ID: 1bg1), crystal structure of PTP1B in complex with compound FMSOA001440b (PDB ID: 5qf5) and GSK3b in complex with inhibitor (PDB ID: 4acc, 4acd, 4acg, and 4ach) from Protein Library Database (https://www.rcsb.org/; accessed on 2 March 2025). Docking simulations were conducted using AutoDock Vina version 1.2.5, with UCHL3, STAT3β, PTP1B, and GSK3β designated as the receptor and docking center. The ligands, (+)-farrerol and (−)-farrerol, were utilized within a grid encompassing a docking range of 70 × 75 × 70. The resulting docking data were saved in PDBQT format to calculate the binding energy (ΔG) between the molecules. The models exhibiting the lowest ΔG values were subsequently imported into PyMol version 3.1.3 for visual analysis.

### 3.8. Stereoselective Growth Inhibition of HT-29 Cells by Farrerol Enantiomers

HT-29 cells were seeded in 96-well plates at a density of 6000 cells per well, and the cells’ formation density reached 80%. HT-29 cells were given a drug-containing medium with different concentrations of farrerol enantiomers (dose concentrations of farrerol were 1 μM, 5 μM, 10 μM, 50 μM, 100 μM, and 500 μM, respectively) and cultured in a CO_2_ incubator for 48 h. At the same time, the normal control group (complete medium added) and the blank group (no cells contained) were set up, and both groups were cultured in a CO_2_ incubator for 48 h without drug administration intervention. Following the conclusion of the culture period, 10 μL of CCK-8 solution was introduced into each well of the experimental groups under dark conditions. The optical density (OD) at 450 nm was subsequently measured using a microplate reader after a 1 h incubation in a CO_2_ incubator, also in the dark. This allowed for the assessment of cell proliferation activity.

### 3.9. Statistical Analysis

Pharmacokinetic analysis was conducted utilizing the Drug and Statistics (DAS) 2.0 software provided by the Chinese Pharmacological Society. To assess the differences in pharmacokinetic parameters, the *IC*_50_ values of the evaluated CYP enzymes and the permeability coefficients between the two farrerol enantiomers, paired sample *t*-tests and independent sample *t*-tests were performed. Statistical significance was defined as * *p* < 0.05 and ** *p* < 0.01. All results are presented as mean values ± standard deviation (SD).

## 4. Conclusions

In conclusion, the stereoselective pharmacokinetics of farrerol enantiomers in rat plasma, liver, and kidney tissues were investigated using an efficient chiral LC-MS/MS method. The (+)-farrerol enantiomer showed significantly greater bioavailability than (−)-farrerol in rat plasma, indicating that the oral pharmacokinetics of the two enantiomers are stereoselective. Notably, in vivo studies demonstrated that chiral inversion of the enantiomers did not occur in rat plasma following oral administration. In contrast, (−)-farrerol has a higher concentration in the rat liver and kidney after oral administration. It is widely recognized that orally administered drugs must overcome three significant barriers to be absorbed into the bloodstream. These include the absorption through the intestinal wall and the enzymatic breakdown in the liver. Consequently, the variations in the in vitro metabolism of specific human CYP enzymes and the in vivo intestinal absorption of farrerol enantiomers in rats were further examined. Among the P450 isoforms examined, there are notable stereoselective differences between (+)-farrerol and (−)-farrerol regarding the inhibition of CYP 1A2, CYP 2C9, CYP 2C19, and CYP 3A4/5 enzymes, which may result in stereoselectivity in liver metabolism and pharmacokinetics between the two enantiomers of farrerol. The absorption of (+)-farrerol and (−)-farrerol in the rat small intestine was not stereoselective within the studied concentration range. Our investigation into the stereoselective binding of farrerol enantiomers to antiproliferative targets (UCHL3, Stat3, PTPN1, and GSK-3β) through molecular docking has revealed significant insights into chiral discrimination at the molecular level. The comprehensive docking analysis demonstrated distinct binding patterns among these targets, highlighting the critical role of stereochemistry in farrerol’s target interactions. Both farrerol enantiomers demonstrated comparable growth inhibition in HT-29 cells, with no significant stereoselectivity observed. To our knowledge, this provides the first evidence focused on the in vivo processes and pharmacological activity of farrero enantiomers.

## Figures and Tables

**Table 1:** Specific optical rotation data of farrerol enantiomers.

Compound	ee	[α]^2^⁰D (MeOH)
(+)-farrerol	98%	+25.93°
(−)-farrerol	98%	−26.13°

**Table 2:** The main pharmacokinetic parameters of (+)-farrerol and (+)-farrerol in rat plasma (*n* = 5).

		C_max_ (ng/mL)	T_max_ (h)	AUC_(0–t)_ (ng/mL*h)	AUC_(0–∞)_ (ng/mL*h)	MRT_(0–t)_ (h)	t_1/2_	F (%)
1	(+)-farrerol	2662.7 ± 226.4	0.08	1464.2 ± 54.5	1552.9 ± 56.0	2.70 ± 0.10	10.7 ± 0.10	/
	(−)-farrerol	1695.0 ± 224.6 **	0.08	1012.4 ± 88.1 **	1195.7 ± 49.7 **	3.48 ± 0.07 *	19.0 ± 3.7 *	/
2	(+)-farrerol	457.2 ± 24.6	1.50	3522.6 ± 146.0	13,885.5 ± 2550.4	5.98 ± 0.14	22.9 ± 2.6	6.4 ± 0.04
	(−)-farrerol	390.0 ± 35.3 *	1.50	1622.6 ± 84.3 **	1837.8 ± 226.9 **	4.65 ± 0.06	3.5 ± 0.4 **	4.3 ± 0.7 *
3	(+)-farrerol	287.0 ± 24.6	3.00	2399.1 ± 102.3	6870.3 ± 511.4	6.27 ± 0.27	19.2 ± 2.0 **	8.7 ± 0.7
	(−)-farrerol	80.5 ± 7.5 **	1.00 *	428.3 ±88.2 **	1341.8 ± 337.6 **	5.61 ± 0.56	8.5 ± 1.5 **	2.3 ± 0.05 **
4	(+)-farrerol	1177.4 ± 84.5	5.00	17,593.5 ± 1273.3	22,122.4 ± 1894.1	9.12 ± 0.18	10.4 ± 0.4	/
5	(−)-farrerol	520.9 ± 91.7 ^#^	2.00 ^#^	6746.8 ± 298.7 ^#^	8572.4 ± 221.0 ^#^	10.84 ± 0.47	12.0 ± 0.5	/

Note: * *p* < 0.05, ** *p* < 0.01 indicate significant difference as compared to (+)-farrerol; compared; ^#^ *p* < 0.01 indicate significant difference as compared to i.g. (75 mg/kg) of (+)-farrerol. 1-i.v. of farrerol racemate (4 mg/kg); 2-i.g. of farrerol racemate (150 mg/kg); 3-i.g. of farrerol racemate (75 mg/kg); 4-i.g. of (+)-farrerol (75 mg/kg); 5-i.g. of (−)-farrerol (75 mg/kg).

**Table 3:** The main pharmacokinetic parameters of (+)-farrerol and (+)-farrerol in rat liver and kidney after administering 40 mg/kg farrerol racemate (*n* = 5).

	Liver		Kidney	
	(+)-Farrerol	(−)-farrerol	(+)-Farrerol	(−)-farrerol
C_max_ (µg/g)	103.8 ± 18.05	148.4 ± 2.61 *	3.02 ± 0.35	4.18 ± 0.48 *
T_max_ (h)	2.00	2.00	1.00	1.00
AUC_(0–t)_ (µg/g*h)	976.25 ± 41.60	1200.96 ± 58.92 *	23.93 ± 2.11	28.89 ± 2.53 *
AUC_(0–∞)_ (µg/g*h)	994.01 ± 40.84	1217.62 ± 65.03 *	25.70 ± 2.09	30.31 ± 2.47 *

Note: * *p* < 0.05 indicates a significant difference as compared to (+)-farrerol.

**Table 4:** The *IC*_50_ value of positive inhibitor, (+)-farrerol and (−)-farrerol.

CYP Subtype	Marker Substrate	Positive Inhibitor		(+)-Farrerol	(−)-farrerol
			*IC*_50_/μmol·L^−1^	*IC*_50_/μmol·L^−1^	*IC*_50_/μmol·L^−1^
1A2	phenacetin	*β*-naphthoflavone	0.129	0.588	1.02 **
2C9	diclofenac	sulfaphenazole	0.219	1.97	1.57 *
2C19	*S*-mephenytoin	Benzenifanol	0.106	1.61	2.07 *
2D6	dextromethorphan	quinindium	0.0744	>50	>50
3A4/5(M)	midazolam	ketoconazole	0.0104	>50	20.9 **
2A6	coumarin	Tranylcypromine	0.383	>50	>50
2E1	chlorzoxazone	4-methylpyrazole	1.46	>50	>50

Note: * *p* < 0.05, ** *p* < 0.01 indicate significant difference as compared to (+)-farrerol.

**Table 5:** The K_a_ and *P*_eff_ values of (+)-farrerol and (−)-farrerol in different regions with and without verapamil (*n* = 3).

	Concentration of Farrerol Racemate/μg·mL^−1^	*P*_eff_ (×10^−1^ cm·min^−1^)		K_a_ (×10^−1^ min)	
		(+)-Farrerol	(−)-farrerol	(+)-Farrerol	(−)-farrerol
Duodenum	66.00	1.32 ± 0.03	1.33 ± 0.03	1.47 ± 0.02	1.47 ± 0.02
	132.00	1.16 ± 0.11	1.17 ± 0.10	1.87 ± 0.43	1.87 ± 0.43
	180.00	1.30 ± 0.14	1.33 ± 0.11	2.04 ± 0.28	2.04 ± 0.28
	132.00+ verapamil	1.42 ± 0.17 *	1.43 ± 0.17 *	1.79 ± 0.45	1.79 ± 0.45
Jejunum	66.00	1.16 ± 0.23	1.17 ± 0.24	1.92 ± 0.10	1.92 ± 0.10
	132.00	0.73 ± 0.03	0.74 ± 0.05	0.71 ± 0.17	0.71 ± 0.17
	180.00	1.03 ± 0.06	1.07 ± 0.10	1.14 ± 0.18	1.14 ± 0.18
	132.00+ verapamil	1.12 ± 0.10 *	1.12 ± 0.10 *	1.16 ± 0.11 *	1.16 ± 0.11 *
Ileum	66.00	1.12 ± 0.22	1.14 ± 0.22	2.05 ± 0.06	2.05 ± 0.06
	132.00	0.87 ± 0.08	0.89 ± 0.10	0.84 ± 0.14	0.84 ± 0.14
	180.00	0.79 ± 0.04	0.78 ± 0.07	1.48 ± 0.39	1.48 ± 0.39
	132.00+ verapamil	0.77 ± 0.11	0.77 ± 0.11	0.78 ± 0.28	0.78 ± 0.28
Colon	66.00	1.09 ± 0.37	1.12 ± 0.41	1.73 ± 0.81	1.74 ± 0.81
	132.00	0.93 ± 0.09	0.95 ± 0.10	0.83 ± 0.27	0.83 ± 0.27
	180.00	1.04 ± 0.12	1.12 ± 0.17	1.21 ± 0.16	1.21 ± 0.16
	132.00+ verapamil	1.41 ± 0.23 *	1.42 ± 0.23 *	1.16 ± 0.36 *	1.16 ± 0.36 *

Note: * *p* < 0.05 indicates a significant difference compared to 132 μg·mL^−1^ farrerol racemate without verapamil.

**Table 6:** Study on molecular docking binding energy of farrerol enantiomers (kcal/mol).

Antiproliferative Target	Receptor	Ligands	Residues Involved in Hydrogen Bond Formation	ΔG	|ΔΔG|
UCHL3	7yv4	(+)-farrerol	ARG215, ASP216	−3.63	7.57
		(−)-farrerol	LYS187, ARG215	−11.20	
Stat3	1bg1	(+)-farrerol	LEU706, ALA703	−4.30	0.14
		(−)-farrerol	LEU706	−4.44	
PTPN1	5qf5	(+)-farrerol	GLU4, LYS279	−18.57	1.30
		(−)-farrerol	LYS279	−19.87	
GSK-3β	4acc	(+)-farrerol	ASP200, GLN185	−6.82	1.64
		(−)-farrerol	VAL135	−8.46	
	4acd	(+)-farrerol	ILE62, VAL70, THR138, GLN185, LEU188	−6.41	2.28
		(−)-farrerol	ASP133, VAL135	−8.69	
	4acg	(+)-farrerol	ILE62, VAL70, VAL110, THR138, LEU188	−6.17	2.47
		(−)-farrerol	ILE62, VAL70, TYP134, LEU188	−8.64	
	4ach	(+)-farrerol	GLN185, ASP200	−6.47	1.25
		(−)-farrerol	ILE62, LEU132, TYP134, LEU188	−7.72	

Note: ΔG and |ΔΔG| refer to the binding energy and the difference between the binding energies, respectively, in kcal/mol.

## Data Availability

The original contributions presented in this study are included in the article/Supplementary Material. Further inquiries can be directed to the corresponding author(s).

## Supplementary Materials

The following supporting information can be downloaded at: https://www.mdpi.com/article/10.3390/molecules30092038/s1, Figure S1: Typical LC-MS/MS ion chromatograms of (+)-/(−)-farrerol and internal standard; Table S1: The calibration curves of farrerol enantiomers; Table S2: The accuracy and precision of the method for the determination of farrerol enantiomers; Table S3: The extraction recovery and matrix effect of farrerol enantiomer.
