# Supplementary material for: Stereoselectivity of In Vivo Processes and Bioactivity of Farrerol Enantiomers
Source: Molecules. 2025 May 3;30(9):2038. doi: 10.3390/molecules30092038 (PMC12073438; doi:10.3390/molecules30092038)
Supplement: Supplementary file 1 [file molecules-30-02038-s001.zip › molecules-3572207-supplementary.pdf]

Stereoselectivity of In Vivo Processes and Bioactivity of Farrerol Enantiomers

Lirong Chen †, Tang Yan †, Dongting Huang, Wei Xu, Yongjing Liu *, Xiaoying Wang * and Hua Li

Institute of Structural Pharmacology & TCM Chemical Biology, College of Pharmacy, Fujian University of Traditional Chinese Medicine, Fuzhou 350122, China; clr1183@163.com (L.C.); 17684061023@163.com (T.Y.); h3315419773@163.com (D.H.); 2000017@fjtcm.edu.cn (W.X.); 2022041@fjtcm.edu.cn (H.L.)

* Correspondence: 2009053@fjtcm.edu.cn (Y.L.); wangxy623@yeah.net (X.W.)

† These authors contributed equally to this work.

1. Methods validation

The selectivity is assessed by comparing the chromatograms of blank plasma or tissue samples with those of samples added to the analyte and the IS. Under the optimum HPLC-MS/MS conditions, the representative chromatograms of blank plasma and liver and kidney tissues (A), standard plasma samples (liver and kidney tissues) spiked with rac-farrerol and IS with the concentration of 48.500 ng·mL-1(B), as well as the actual plasma and liver and kidney tissues after administration (C) were displayed in Figure S1. It can be seen that there were no peaks eluted at the retention time of (+), (-)-farrerol, and IS in blank plasma and tissue samples, indicating that endogenous interference did not affect the detection of analytes and the IS.

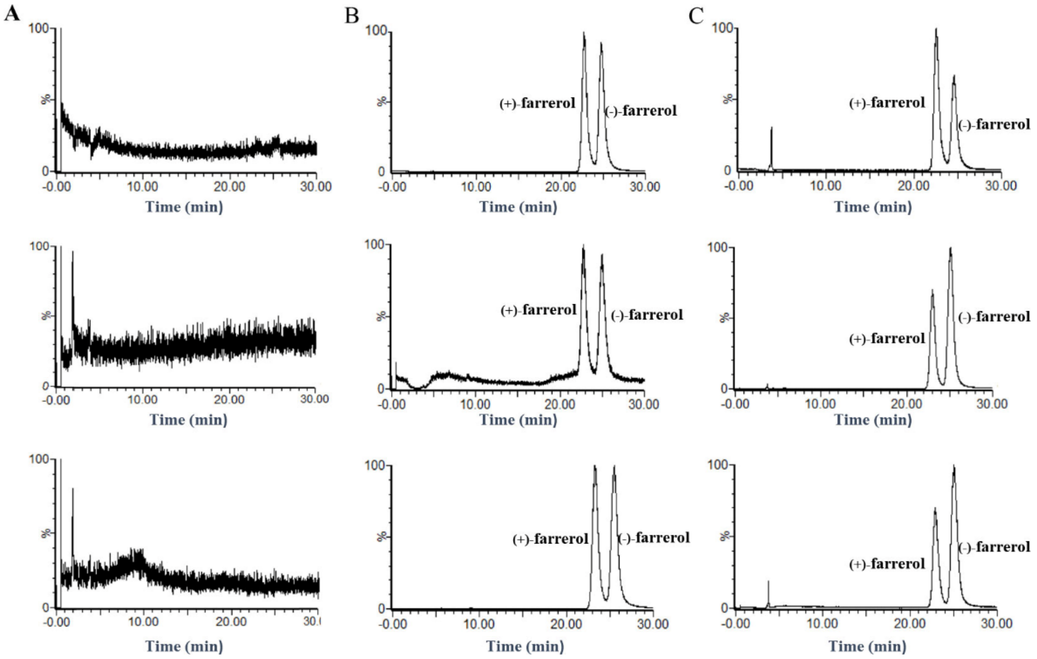

Figure S1. Typical LC-MS/MS ion chromatograms of (+)-/(-)-farrerol and internal standard in (A) blank rat plasma or tissue, (B) plasma or tissue spiked with farrerol racemate and IS, (C) actual plasma and liver and kidney tissues after administration of farrerol le racemate.

Linearity was evaluated by establishing a calibration curve of target analytes at different concentration levels in three different analytical batches. The calibration curves were plotted as peak area ratios of (each farrerol enantiomer / IS) (y) versus the concentration of a single enantiomer in plasma or tissues (x). The calibration curves and correlation coefficient of the two farrerol isomers in plasma and tissues are shown in Table S1. As evident from Table S1, the R2 was greater than 0.995 for each

regression curve, indicating the linearity was acceptable.

Table S1 The calibration curves of farrerol enantiomers

	analyte	regression equation	*r*	linearity range /ng·mL^{-1}
Plasma	(+)- farrerol	$y = 0.0087\,x - 0.0024$	0.9981	9.76～5018.40
	(-)-farrerol	$y = 0.0091\,x + 0.0016$	0.9981	10.16～5181.60
Liver	(+)- farrerol	$y = 0.0072\,x - 0.0065$	0.9988	9.76～6247.50
	(-)-farrerol	$y = 0.0071\,x - 0.0023$	0.9976	10.16～6502.50
Kidney	(+)- farrerol	$y = 0.0088\,x + 0.0027$	0.9942	9.76～2499.00
	(-)-farrerol	$y = 0.0090\,x + 0.0070$	0.9948	10.16～2601.00

The precision and accuracy of the proposed method in plasma and tissues were investigated by analyzing QC samples at three different levels in six replicates within one day (intra-day) and during three consecutive days (inter-day). The results of relative error (RE) and the relative standard deviation (RSD) which were presented to reveal the accuracy and precision of the proposed method were exhibited in Table S2, The results verified that the stated stereoselective method was reproducible and reliably adopted in the determination of (+)-farrerol and (-)-farrerol in plasma and tissues.

Table S2 The accuracy and precision of the method for the determination of farrerol enantiomers(n=3)

	analyte	Concentration /ng·mL^{-1}	intra-day		inter-day	
			RSD/%	RE/%	RSD/%	RE/%
Plasma	(+)- farrerol	9.76	2.19	89.02	3.39	89.99
		1249.50	1.29	92.56	2.84	91.22
		3748.50	1.70	99.58	3.14	99.31
	(-)-farrerol	10.16	1.16	87.99	2.51	87.21
		1300.50	1.06	89.66	1.74	88.13
		3886.27	0.92	100.19	2.96	99.86
Liver	(+)-farrerol	9.76	1.04	98.64	1.90	97.51
		1249.50	3.26	105.51	2.80	100.54
		4685.62	2.27	97.08	2.75	96.43
	(-)-farrerol	10.16	2.55	95.15	1.36	93.24
		1300.50	0.79	106.82	0.76	103.03
		4876.88	1.20	96.97	1.14	94.65
Kidney	(+)- farrerol	9.76	0.96	87.16	2.34	86.27
		624.75	3.02	101.95	3.26	100.05
		1874.25	2.36	99.27	1.57	98.43
	(-)-farrerol	10.16	1.36	86.70	2.05	85.65
		650.25	2.13	102.78	1.69	101.26
		1950.75	0.19	99.02	0.82	98.77

To investigate the recovery of each farrerol enantiomer and IS in rat plasma and tissue samples, three quality control concentrations of samples at low, medium, and high levels were prepared and pretreated in six replicates. The extraction recovery was calculated by comparing the individual peak

area of the farrerol enantiomer and the IS of extracted QC samples (spiked before extraction) with that of unextracted plasma and tissue samples(spiked after extraction). The matrix effect was evaluated by comparing the peak areas of each farrerol enantiomer and IS, which were measured by analyzing six different blank biosamples spiked with standards after extraction, with those obtained by neat standard solutions at the same concentration. All data are displayed in Table S3, which confirms that the extraction procedure of farrerol enantiomers and IS from rat plasma and tissue was valid, as well as endogenous compounds co-eluted, could not produce obvious ion suppression or enhancement.

Table S3 The extraction recovery and matrix effect of farrerol enantiomer($x \pm$ s， n=3)

analyte		Concentration /ng·mL^{-1}	extraction recovery /%	matrix effect /%
Plasma	(+)-farrerol	9.76	86.15± 4.22	100.68± 1.99
		1249.50	88.98± 5.52	98.78± 0.77
		3748.50	87.40± 0.31	99.14± 5.38
	(-)-farrerol	10.16	93.65± 5.15	95.39± 0.95
		1300.50	100.79± 4.96	104.81± 8.19
		3886.27	99.12± 1.96	97.78± 1.25
Liver	(+)-farrerol	9.76	86.15± 2.68	102.90± 6.77
		1249.50	79.53± 0.98	98.24± 1.41
		4685.62	104.63± 0.54	99.20± 2.04
	(-)-farrerol	10.16	98.41± 0.93	98.45± 1.97
		1300.50	83.33± 2.54	99.30± 2.87
		4876.88	78.95± 1.51	100.63± 4.10
Kidney	(+)-farrerol	9.76	95.38± 0.78	97.95± 1.13
		624.75	84.25± 0.88	98.47± 1.22
		1874.25	102.31± 4.79	99.72± 3.09
	(-)-farrerol	10.16	104.76± 3.87	99.00± 1.82
		650.25	84.92± 0.07	100.71± 3.75
		1950.75	85.74± 3.27	99.67± 3.41

The stability of the tested ferrorol enantiomers and the IS was assessed by analyzing six plasma and tissue samples at low, medium, and high QC levels. The different sample storage and handling environment conditions were as follows: (a) short-term stability: stored at room temperature for 24 h; (b) freeze-thaw stability: undergoing -20°C freezer for 12 h and 25°C ambient temperature three times. (c)long-term stability: enantiomers were frozen at -20°C for one and two weeks. The results of stability show that the RSD value in the plasma and tissue samples did not exceed 4.92% for each ferrorol isomer, indicating that the ferrorol enantiomers were considered to be stable under the tested conditions.
